# The Design and Evaluation of a Simulation Tool for Audiology Screening Education: Design Science Approach

**DOI:** 10.2196/47150

**Published:** 2025-02-20

**Authors:** John Gerdes, Benjamin Schooley, Dakota Sharp, Juliana Miller

**Affiliations:** 1 Department of Integrated Information Technology University of South Carolina Columbia, SC United States; 2 College of Engineering and Computing Brigham Young University Provo, UT United States; 3 School of Medicine University of North Carolina Durham, NC United States; 4 Department of Communication Sciences and Disorders University of South Carolina Columbia, SC United States

**Keywords:** design science, audiology, simulation, hearing screening, framework, speech pathology, training

## Abstract

**Background:**

The early identification of hearing loss and ear disorders is important. Regular screening is recommended for all age groups to determine whether a full hearing assessment is necessary and allow for timely treatment of hearing problems. Procedural training is needed for new speech-language pathology students as well as continuing education for those trained to perform this screening procedure. Limited availability and access to physical training locations can make it difficult to receive the needed training.

**Objective:**

The aims of this study were to (1) develop a new hearing screening simulation software platform and (2) assess its effectiveness in training a group of graduate-level speech-language pathology students in hearing screening procedures.

**Methods:**

An audiology simulator modeled after the commercial Grason-Stadler GSI39 combination audiometer and tympanometer device was developed to serve as a precursor to traditional face-to-face clinical instruction. A description of the simulator development process, guided by a design science approach, is presented. The initiation phase established the initial criteria for the simulator design. This was followed by an iterative process involving prototype development, review, and critique by the clinical faculty. This feedback served as input for the subsequent iteration. The evaluation of the final prototype involved 33 speech-language pathology graduate students as part of an introductory audiology class. These students were randomly assigned to control (receiving in-person instruction) and test (in-person instruction and simulation tool use) groups. Students in both groups were subsequently evaluated as they performed audiology screenings on human participants and completed a 25-item pretest and posttest survey. Nonparametric Mann-Whitney *U* tests were conducted on the mean differences between pretest and posttest ordinal survey response data to compare the control and intervention groups.

**Results:**

The results indicated that the students who used the simulation tool demonstrated greater confidence in their ability to (1) explain hearing screening procedures to a child (*P*=.02), (2) determine whether otoscopy results are normal (*P*=.02), and (3) determine whether otoscopy results are abnormal (*P*=.03). Open-ended responses indicated that the students found that the hands-on experience provided by the simulator resulted in an easy-to-use and useful learning experience with the audiometer, which increased their confidence in their ability to perform hearing screenings.

**Conclusions:**

Software-based education simulation tools for audiology screening may provide a beneficial approach to educating students and professionals in hearing screening training. The tool tested in this study supports individualized, self-paced learning with context-sensitive feedback and performance assessment, incorporating an extensible approach to supporting simulated subjects.

## Introduction

### Background

A national study in the United States found that 14.9% of children ranging in age from 6 to 19 years had low- or high-frequency hearing loss in one or both ears [1]. Early identification, diagnosis, and intervention are beneficial, improving the likelihood that children will develop effective communication and language skills and achieve successful learning outcomes [2]. For this reason, hearing screenings should be carried out regularly to identify problems early. To accomplish this, health care students and personnel must receive adequate training on how to perform these screenings and appropriately refer patients for evaluation [3].

The American Speech-Language-Hearing Association guidelines for audiological screening [4] specify procedures for completing hearing loss screenings, along with recommendations for screening the outer and middle ears, collecting an optional case history, performing a visual examination (otoscopy), and conducting acoustic immittance testing (tympanometry). Those who do not pass a hearing screening (the term “fail” is not used) are typically referred to a licensed audiologist for more detailed hearing testing or to a physician for any medical concerns. The screener can also refer the individual to both an audiologist and physician when the results warrant such action.

The American Speech-Language-Hearing Association recognizes the use of standardized patients and simulation technologies as alternatives to traditional clinical education methods [5]. The use of clinical simulation increased significantly during the COVID-19 pandemic because many practicum sites became inaccessible to students. Simulation is increasingly being used in clinical training and has been found to improve clinical knowledge and skills, self-confidence, communication skills, empathy, critical thinking abilities, leadership, and situation management among students [6-11]. Simulation has been found to improve the confidence in both knowledge and skills of audiology and speech-language pathology graduate students during training [12,13]. A survey of clinical educators found that simulation was a useful learning tool, provided a quality control measure, was an aid in professional development, and exposed students to clinical pathologies that were not routinely encountered [13]. Barriers to the use of simulation include faculty lacking training with simulators, limited funding to purchase simulator technology, and a lack of resources such as time and space [13].

Training speech-language pathology students to perform hearing screenings is an important undertaking. Students must learn how to interact with children and adults, properly work through the assessment protocol, and learn how to operate the equipment.

Studies have shown simulation to be effective in audiology education and training [14-16]. Simulation can help transfer theory to practice in an integrated teaching and learning model [17]. Simulators can present a full range of patient cases that represent real-world clinical diagnoses. These simulated cases present patients with symptoms that must be detected and diagnosed by the student. However, the use of simulation in audiology education is still in its infancy compared to some other fields, such as nursing and medicine [3].

It can be challenging for speech-language pathology students to obtain the necessary audiology training due to limited practicum opportunities. One alternative is to use a commercial simulator application, such as Otis–the virtual patient [18], which is a simulator designed for use in the education of audiologists. Many of these commercial simulators are designed to train students in conducting full audiological evaluations, rather than teaching hearing screening skills that follow an abbreviated protocol. However, these simulators can be cost prohibitive for training speech-language pathology students. Our goal was to deliver a realistic simulation tool providing a realistic representation of the controls found on a commercial testing instrument that students would work with when in the field. This design allows students to practice and develop testing skills without having to travel to a testing center. It also exposes them to a broad array of clinical cases and provides feedback on their performance. These goals became particularly relevant during the COVID-19 pandemic, when participating in live practicum experiences became difficult.

Prior research has explored the use of pure-tone audiometry simulators [19-23]. One system incorporated multilingual capability, improving accessibility for non-English speakers [21]. Audiology training using virtual reality was found to yield positive results compared to traditional training [11]. There are also commercially available simulation tools that are useful for training audiology skills. AudSim Flex [24] offers basic and advanced simulators for audiology students to learn pure-tone and bone conduction testing, both masked and unmasked. Otis–the virtual patient [18] realistically simulates the behavior of the patient and detects possible user errors immediately. It supports both pure-tone and screening training and guides the learner through exercises at several levels of difficulty, while providing useful help.

There are similarities between the hearing screening simulator (HSS) we developed and existing simulation tools. All incorporate a set of simulated subjects that present a range of hearing disorders, provide background narratives of the subjects to provide context, and model human behavior or actual subjects to improve realism. Most tools allow students to perform a simulated assessment and collect data that are then plotted on a standard audiogram, with the simulator assessing and critiquing the students’ analysis and conclusions.

The HSS differs from other simulators in 2 important ways. First, the HSS provides a digital twin—a realistic representation of the Grason-Stadler GSI39 combination audiometer and tympanometer device [25]—whereas other simulators use a generic design. By modeling the actual instrument interface, the HSS allows the student to become familiar with the instrument layout and practice working with the modeled device’s controls. Second, the HSS is designed for the simpler hearing screening protocol rather than full audiological testing. The protocols are different; therefore, the feedback and assessment verification must be tailored accordingly. Full audiological testing is outside the scope of practice for speech-language pathology students.

A design science approach was adopted for the development of the HSS. Initial design specifications were first established, followed by an iterative process involving prototype development, review, and critique by the clinical faculty, with the feedback used to refine the next iteration. After several iterations, the final prototype was tested to assess whether students felt that the simulator was useful in learning how to administer hearing screenings and to determine their confidence in their skills after using the simulator.

### Objectives

The aims of this study were to (1) develop a new hearing screening simulation software platform and (2) assess its effectiveness in training a group of speech-language pathology students in hearing screening procedures. This work’s contributions include a framework for developing a simulation of an existing process (hearing screening testing) and the description of a unique artifact with a user interface that closely emulates an existing commercial tool used to perform hearing screenings. This artifact supports an individualized, self-paced learning environment incorporating context-sensitive feedback and performance assessment. While further testing is needed, initial testing indicates that the HSS shows promise as a training aid for speech-language pathology students.

## Methods

### Overview

This research follows a design science methodology consisting of (1) problem identification and motivation, (2) the definition of the objectives for a solution, (3) design and development, (4) demonstration, (5) evaluation, and (6) communication [26,27]. We identified and translated design requirements for an audiology simulator into design components. We then classified these functional components in the application into design principles and features for designing a health care simulation app for clinical education and training. An abbreviated description of the design phase is provided in the next subsection, offering context for the evaluation phase of the study. For a more detailed description of the development process, the interested reader is directed to the conference paper [28]. For the evaluation of the simulation tool, participants were randomly separated into control (did not use the simulator) and test (used the simulator) groups, with both groups completing a pretest and posttest survey. Survey responses were analyzed using the nonparametric Mann-Whitney *U* test, which is appropriate for ordinal data analysis when a normal distribution cannot be assumed. Four qualitative survey question responses were also collected and analyzed. Moreover, audiology instructors assessed the skill levels of the student participants. Additional details on the analysis can be found in the Evaluation Study subsection.

### The Simulation Tool’s Conceptual Framework, Requirements, and Design

The simulation tool was designed to train speech-language pathology students on the proper use of audiometer equipment, how to administer a hearing screening test, and how to interpret the results. The tool’s clinical and administrative functions, which are summarized in Textbox 1, served as the design criteria for the HSS. The clinical requirements reflect the data typically collected and presented during a traditional training session using commercial equipment. The operational requirements include additional features that enable a web-based digital learning experience, such as calculating student grades, and therefore represent value-added aspects of the simulator.

The HSS is designed to simulate 3 clinical procedures used in hearing screening and audiological evaluations. The otoscopic examination is a visual procedure used to assess the condition of the external auditory canal and tympanic membrane. Tympanometry tests how well the subject’s eardrum moves and determines the volume of the ear canal and the pressure in the middle ear space. The audiometer presents a series of tones to the subject based on the appropriate protocol, and the subject indicates whether they hear the tone by raising their hand. For screening tests, tones are presented at a fixed level considered “normal hearing” (20 dB hearing level), in contrast to a typical diagnostic hearing test where multiple levels are tested to determine the subject’s hearing threshold at each frequency (ie, the lowest level the subject can hear). The HSS uses standardized subjects with known auditory hearing profiles, along with sample otoscopic images and tympanometry results for both ears.

**Figure 1:**
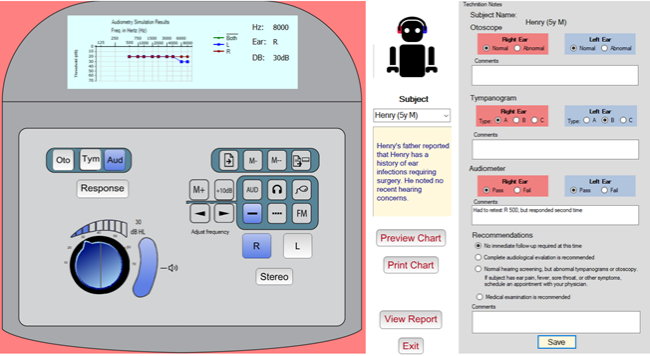
The hearing screening simulator user interface. (A) The simulated audiometer device. (B) The panel that provides the simulated subject’s details, with the avatar responding to tone stimulus. (C) Area for the technician to enter results and comments.

The simulator user interface consists of 3 panels. Figure 1A shows the simulated audiometer device, which was modeled after the Grason-Stadler GSI39 combination audiometer and tympanometer device [25]. The upper portion of the unit contains a graphic display that shows either the tympanogram or audiogram depending on the testing mode selected. The lower portion of the unit provides the controls used to perform the screening. These controls and their purpose are described herein. Figure 1B portrays the panel that provides information about the subject, as well as the command buttons to show and print a larger version of the audiogram chart, and displays the debriefing report that summarizes the students’ performance on the simulation. Figure 1C shows the technician notes panel. It allows students to record their findings and include their observations, as is typical in a traditional audiological screening.

**Required clinical and operational functionalities of the hearing screening simulator.**

**Clinical requirements**

- Authenticate and authorize users
- Provide a graphical user interface similar to that of standard audiology screening hardware-based systems
- Simulate 3 audiometry screens: video otoscopy, tympanometry, and audiometry
- Configure audiometer settings for a specific subset of frequencies used in hearing screening
- Display output similar to that obtained with commercial devices, including otoscopic images, tympanograms, and audiograms
- Include simulated patients representative of a range of real-world clinical cases

**Operational requirements**

- Provide a web-based platform
- Track and report time spent on the simulation
- Document and print a record of the simulation session
- Include automatic grading and feedback on students’ performance
- Provide a full-featured audiometer with additional features beyond those needed for the hearing screening learning experience but present in commercial units
- Log all simulation screenings to permit reporting of an individual student’s simulation history and enable cross-sectional data analysis across multiple students’ results

The HSS is equipped with 10 simulated patients, with the capability to expand this list. Each patient has a short case history, which is presented to the student when the subject is selected, providing the subject’s age and sex and a brief biographical background related to any hearing concerns. The 10 artificial subjects represent different audiological conditions and pathologies, with audiometry, tympanometry, and otoscopy results consistent with their condition. One child and 1 adult standard patient with normal results were included. To obfuscate the identities of these simulated patients and create the illusion of having a much larger subject base, the profile information is randomized each time the program is run. Subject’s sex is randomly assigned, age is adjusted slightly, the subject’s name is randomly selected from a list of 100 diverse names, and the backstory is modified to match the new subject profile. This feature expands the number of possible simulated patient profiles to >1000 unique profiles. This prevents students from circumventing the training by using results from earlier simulations and also enables the assessment of a student’s precision when presented with multiple subjects derived from the same base simulated patient.

To perform the training, the student selects a standardized subject and reads the brief biographical sketch provided. The simulated screening test is then performed. The student must correctly set up the audiometer and work through the testing procedure. The otoscopic examination is simulated by selecting either the right or left ear and pressing the “Oto” button, which displays the subject’s right or left otoscopic image, depending on which ear is specified. Similarly, the student simulates the tympanometry test by selecting the ear and pressing the “Tymp” button, displaying the subject’s tympanometry result. To simulate the auditory test, the student selects the ear being tested, the tone level (20 dB hearing level) for the test, and then the series of frequencies specified by the testing protocol.

As the student proceeds through the simulation, they are asked to record their observations and patient results in the technician notes panel. There are note sections for each of the 3 screening procedures (otoscopy, tympanometry, and audiometry). The student is asked to provide the result for each ear and to add comments and observations related to the screening. Recording this information allows the simulator to assess the student’s performance. The system knows which result is correct based on the known profile of the subject being screened. Taking into consideration the 3 individual screening procedures, the student must also make a final recommendation regarding the patient’s subsequent follow-up and add a related comment. The student’s final recommendation is automatically graded and reported by the simulator, immediately providing appropriate feedback as needed. The patient cases, associated correct results, and correct recommendations were defined by the study clinical team and implemented in the application database.

**Figure 2:**
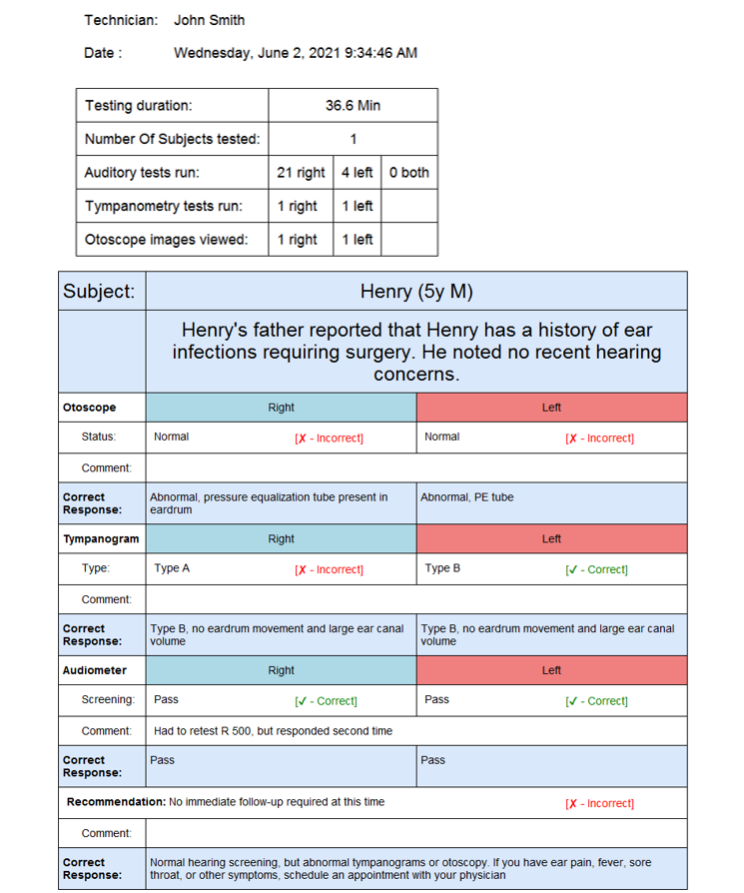
Simulation report summary displaying student performance in administering a hearing screening to a simulated client. It includes session metadata and a simulation debriefing report that provides the students’ responses, notes, and correct results.

Once the student completes the screening and enters their session notes, the data are saved to a database, and a session report is generated (Figure 2). This report serves as a starting point for a debriefing that would occur between a facilitator and the student. It provides metadata related to the simulation, including the technician’s name (ie, the student’s name), the subject’s name, the duration of the testing portion of the simulation, the screening procedures run by the student during the evaluation, and the total time spent on the simulation session. This information allows the simulator to perform validity checking of the training session, including whether the proper procedure was followed and the proper tests were run. The session report provides information to the simulation facilitator about the steps completed by the student during the session. Any shortcuts taken by the student during the session would be noted in the report. The report also displays the student’s results along with the correct results, with an explanation justifying the results. This provides the student with immediate feedback on their performance as well as their performance over time. Reports can be printed or saved as a PDF file to serve as required documentation of their clinical training.

### Evaluation Study

#### Student Participant Sampling, Intervention Procedure, and Data Collection

All 33 first-year speech-language pathology graduate students enrolled in an Introduction to Audiology class participated in this pilot study. The study started with 34 students, but since one student in the text group failed to submit the posttest survey they were dropped from the analysis resulting in 33 subjects. This is a required course in the graduate Speech-Language Pathology curriculum.

All participants received the same lecture on hearing loss concepts and performing hearing screening assessments. The 33 students were randomly assigned to either the control (n=16, 48%; all female) or test (n=17, 52%; all female) group by listing all participants in Microsoft Excel and using the built-in randomization function. The control group received brief in-person training on equipment operation before being evaluated when they conducted their live, in-person audiology screening. The test group received the same in-person equipment operation introduction but used the simulation tool before being evaluated doing their live audiology screening. Students in each group were divided into 2-member teams, except for 1 group of 3 in the test group. Each student team alternated between roles, acting as a test subject (ie, patient) and as the test evaluator. The practice of students acting as both subject and evaluator is common in small class settings for audiology training. Approximately half of the students in the control group (16/33, 48%) and those in the test group (17/33, 52%) had previous hands-on experience with a hearing screening audiometer. Randomization included sorting students into an approximately equal number of pairings of “no previous experience,” “1 partner with previous experience,” and “both partners with previous experience.”

Participants in the test group received supplementary training with the simulation tool. They were introduced to the simulator and given approximately 15 minutes to work through approximately 5 cases with their partner. A 25-item questionnaire was administered to all students to assess their confidence in their skills both before and after performing the live hearing screening. This means that the control and test groups completed the questionnaire both before and after the intervention. The questionnaire was created by the audiology faculty to gauge students’ knowledge and confidence in administering and interpreting hearing screenings. A 5-point Likert scale was used. The response options and corresponding coding values were *strongly disagree* (1), *disagree* (2), *neither disagree nor agree* (3), *agree* (4), and *strongly agree* (5). A set of 7 to 9 questions were asked about each assessment mode (otoscopy, tympanometry, and audiometry). The test group was also asked 4 additional free-response questions in the same questionnaire to assess their perceptions of the simulation tool. A total of 16 surveys were completed by the control group and 17 completed by the test group. One test group survey was excluded from analysis because it represented the second of 2 surveys completed by the same participant, each providing identical feedback. Thus the analysis use 16 surveys in each group.

#### Student Participant Survey Data Analysis

A nonparametric analysis was conducted using the Mann-Whitney *U* test to compare differences between the groups at baseline (ie, before the test) and after the intervention (ie, after the test). The goal was to assess the change in the test and control groups’ pretest versus posttest survey responses. The Mann-Whitney *U* test is a nonparametric method suitable for analyzing ordinal data when a normal distribution cannot be assumed. In this case, the ordinal variables were responses to survey questions measured on a Likert scale. This method is also appropriate when the groups being analyzed represent 2 independent samples (test and control groups created via randomization). The null hypothesis is that the means of each group are identical, meaning that all groups come from the same distribution. The alternative hypothesis is that at least 1 of the groups has a different mean, meaning that at least 1 group comes from a different distribution than the others. The test was corrected for tied ranks.

It was expected that using the simulation training tool together with in-person training would be perceived by the students to be at least as effective as, and possibly an improvement over, in-person–only training using commercial audiology equipment. Although some indication of improvement was expected, statistically significant differences between the groups were not expected for most questionnaire items between the test and control groups. All statistical analyses were conducted using a standard, commercially available statistical software tool (NCSS 2021; NCSS Statistical Software). A thematic analysis of qualitative responses was conducted to produce categorical findings, which were then discussed.

#### Student Qualitative Data Collection and Analysis

As part of the posttest survey administered to the test group, 4 free-response questions were included regarding their impressions of using the simulator tool. Qualitative responses were typed, and we conducted a thematic analysis across the responses, which were then grouped, summarized, and counted.

#### Instructor Evaluation of Student Hearing Screening Assessment Technique

A live assessment of the students’ clinical techniques was completed using commercial audiometry units, with peers role-playing as patients. Certified instructors supervised the assessment, observing and grading the students’ performance on otoscopy, tympanometry, and hearing screening techniques on a scale ranging from 1 to 5. Scores were compiled, averaged, and compared across the control and test groups.

### Ethical Considerations

The institutional review board of the University of South Carolina determined that this research did not involve human subjects. All participants received a verbal and written description of the study and consented to participate. Participants’ identities were kept confidential and anonymized before data analysis. No compensation was provided for participation in the study.

## Results

### The Simulation Tool’s Conceptual Framework

#### Overview

In a design science study, the objective is to assess how well the created artifact, in this case, the HSS, meets the target operational requirements. In this study, we focused on assessing how well the tool performed in delivering the necessary training on the audiometer equipment. The results are presented in the following subsections.

#### Evaluation Study

#### Likert Scale Survey Results

The test group showed higher posttest mean scores and greater percentage change than the control group across all 3 test component groupings: otoscope, tympanometer, and hearing screening (Table 1).

**Table 1:** Multifactor assessment of student confidence change resulting from the use of the simulation tool. Pretest and posttest values represent the averages for both control and test groups.

	Control group (n=16)			Test group (n=17)		
	Pretest score, mean (SD)	Posttest score, mean (SD)	Change (% change)	Pretest score, mean (SD)	Posttest score, mean (SD)	Change (% change)
Otoscope (7 factors)	3.59 (0.41)	3.90 (0.63)	0.31 (8.64)	3.73 (0.58)	4.28 (0.55)	0.55 (14.75)
Tympanometer (9 factors)	3.63 (0.32)	4.28 (0.50)	0.63 (17.35)	3.79 (0.70)	4.62 (0.44)	0.83 (21.90)
Hearing screening (9 factors)	4.01 (0.56)	4.39 (0.45)	0.38 (9.48)	3.84 (0.59)	4.43 (0.45)	0.59 (15.36)

As shown in Table 2, the responses to the following 3 survey questions showed statistically significant differences between the groups with significance set at *P*<.05: “I am confident in my ability to explain hearing screening procedures to a child” (Mann-Whitney *U*=71.0, n1=n2=16; *P*=.02, 2-tailed *t* test), “I am confident in my ability to determine if otoscopy is normal” (Mann-Whitney n1=n2=16; *P*=.02, 2-tailed), and “I am confident in my ability to determine if otoscopy is abnormal” (Mann-Whitney *U*=74.5, n1=n2=16 *P*=.02, 2-tailed *t* test). For technology pilot studies, findings at the *P*<.10 significance level can also demonstrate important results. In this sense, the responses to the following 2 survey questions showed statistically significant differences between the groups with significance set at *P*<.10: “I am confident in my ability to locate landmarks while completing otoscopy” (Mann-Whitney *U*=86.5, n1=n2=16; *P*=.08, 2-tailed *t* test) and “I am confident which dB level should be tested during a hearing screening” (Mann-Whitney *U*=84.0, n1=n2=16; *P*=.07). The results were not significant across 19 survey questions at the *P*<.10 significance level, underscoring the specific impact of the simulator on the aforementioned confidence measures.

The survey asked whether students had previously conducted an audiology screening test on a human subject before the class audiology training exercise and study. A total of 8 (24%) of the 33 participants in the test group and 6 (18%) of the 33 participants in the control group reported in the affirmative. For this smaller subset of participants, mixed results showed higher posttest mean scores and greater percentage change for the otoscope and tympanometer control groups than the test groups. There was a negative change and percentage change for the combined hearing screening scores for the control group, while the test group had a positive change and percentage change (Table 3). Statistically significant differences were not calculated due to the small sample sizes across the groups.

**Table 2:** Analysis of survey responses grouped by otoscopy, tympanometry, and hearing screening questions, comparing the control and test group mean scores for pre- and postassessment sentiment. The Mann-Whitney U test was used to assess the statistical significance of the change in sentiment between the 2 groups.

Survey questions			Control group					Test group					*P* value
			Preassessment score, mean (SD)		Postassessment score, mean (SD)		Preassessment score, mean (SD)			Postassessment score, mean (SD)			
**Otoscopy**													
	I am confident in my ability to interpret otoscopy	3.63 (0.62)		3.93 (0.80)		3.65 (1.00)			4.06 (0.75)		.74		
	I am confident in my ability to complete otoscopy on a patient	3.19 (0.75)		3.80 (0.77)		3.24 (1.15)			4.18 (0.64)		.39		
	I am confident in my ability to locate landmarks while completing otoscopy	3.44 (0.63)		3.73 (0.70)		3.53 (0.94)			4.35 (0.70)		.08^a^		
	I am confident in my ability to determine if otoscopy is abnormal	3.81 (0.54)		3.60 (1.06)		3.82 (0.73)			4.18 (0.81)		.03^b^		
	I am confident in my ability to determine if otoscopy is normal	3.88 (0.50)		4.00 (0.85)		3.94 (0.66)			4.41 (0.62)		.02^b^		
	I am confident in my ability to determine if otoscopy warrants a referral	3.75 (0.68)		3.93 (0.59)		3.88 (0.49)			4.24 (0.75)		.43		
	I am confident in my understanding of the impact of abnormal otoscopy on hearing screening results	3.38 (0.89)		4.27 (0.46)		4.06 (0.66)			4.56 (0.51)		.32		
**Tympanometry**													
	I am confident in my ability to interpret a tympanogram	3.53 (0.50)		4.40 (0.51)		3.76 (0.97)			4.65 (0.49)		.43		
	I can confidently identify a normal ear canal volume (ECV) on a tympanogram	3.81 (0.54)		4.33 (0.49)		4.06 (0.56)			4.65 (0.49)		.45		
	I can confidently differentiate between tympanogram types	3.75 (0.45)		4.33 (0.62)		3.88 (0.86)			4.65 (0.49)		.76		
	I can confidently identify an abnormal tympanogram	3.81 (0.40)		4.33 (0.62)		4.00 (0.94)			4.71 (0.47)		.66		
	I can confidently identify a normal tympanogram	3.88 (0.34)		4.40 (0.51)		4.24 (0.56)			4.76 (0.44)		.86		
	I can confidently complete a tympanogram on a patient	3.13 (0.81)		4.29 (0.83)		3.18 (1.19)			4.59 (0.51)		.51		
	I am confident in my ability to determine if a tympanometry result warrants a referral	3.69 (0.70)		4.13 (0.52)		3.65 (0.86)			4.53 (0.62)		.12		
	I am confident in my understanding of the impact of abnormal tympanometry on hearing screening results	3.38 (0.81)		4.20		3.76 (0.75)			4.59 (0.51)		.85		
	I am confident in my ability to connect abnormal otoscopy results to abnormal tympanometry results	3.56 (0.51)		3.93 (0.80)		3.59 (1.12)			4.47 (0.62)		.76		
**Hearing screening**													
	I am confident in which frequencies should be tested during a hearing screening	4.31 (0.60)		4.79 (0.43)		4.12 (0.78)			4.65 (0.61)		.24		
	I am confident which dB level should be tested during a hearing screening	4.13 (0.72)		4.53 (0.52)		3.76 (0.90)			4.53 (0.72)		.07^a^		
	I am confident in my ability to explain hearing screening procedures to an adult	3.63 (0.96)		4.27 (0.70)		3.65 (0.86)			4.65 (0.49)		.42		
	I am confident in my ability to explain hearing screening procedures to a child (e.g., conditioned play audiometry)	3.75 (1.00)		4.00 (0.85)		3.38 (0.81)			4.29 (0.85)		.02^b^		
	I am confident in how to present stimuli/tones while completing a hearing screening	3.81 (0.98)		4.36 (0.50)		3.47 (1.07)			4.65 (0.49)		.11		
	I am confident in how to verify a response while completing a hearing screening	4.00 (0.82)		4.27 (0.70)		4.00 (0.87)			4.53 (0.51)		.22		
	I am confident in knowing when a referral is necessary based on hearing screening results	4.06 (0.57)		4.50 (0.52)		3.94 (0.75)			4.41 (0.62)		.60		
	I am confident in knowing which professional to refer to based on hearing screening results	4.38 (0.50)		4.47 (0.52)		4.35 (0.49)			4.47 (0.62)		.75		

^a^Significance set at *P*<.10.

^b^Significance set at *P*<.05.

**Table 3:** Multifactor assessment of student confidence change for students reporting having conducted an audiology screening test on live subjects before the audiology test. Pretest and posttest values represent the average values.

	Control group (n=6)				Test group (n=8)		
	Pretest scores, mean (SD)	Posttest scores, mean (SD)	Change (% change)	Pretest scores, mean (SD)		Posttest scores, mean (SD)	Change (% change)
Otoscope (7 factors)	3.83 (0.40)	4.36 (0.40)	0.53 (13.8)	3.91 (0.49)		4.30 (0.51)	0.39 (10.0)
Tympanometer (9 factors)	3.83 (0.21)	4.67 (0.42)	0.84 (21.9)	4.24 (0.47)		4.75 (0.38)	0.51 (12.0)
Hearing screening (9 factors)	4.56 (0.30)	4.66 (0.41)	−0.10 (−2.2)	4.21 (0.61)		4.67 (0.45)	0.46 (10.9)

#### Qualitative Questionnaire Results

The results of the 4 free-response questions that were administered to the test group regarding their impression of the simulator tool are presented in Table 4. We categorized the responses based on their context similarity (type) and frequency of being mentioned.

The responses to the first question indicate that the students found that the hands-on experience gained by using the simulator provided an easy-to-use and useful learning experience with the audiometer that increased their confidence in their ability to perform hearing screening.

With regard to the second question, the simulator was designed to display an image or chart for a simulated subject, rather than demonstrate how to perform an otoscopic or tympanometric examination. Students wanted more explanation on how to perform these examinations. Others felt that the instructions, in general, could be improved, possibly incorporating pictures or a video. One participant expressed a concern that the simulator design did not resemble an audiometer machine. Given that the simulator was modeled to closely resemble the Grason-Stadler GSI39 combination audiometer and tympanometer device, it is assumed that the student was familiar with a different model.

The most frequent response to the third question was that the simulator should improve training on how to perform otoscopy and tympanometry screenings. It was also suggested that the simulator model the instructions provided by the technician to the subject. Future software iterations will address these suggestions.

The responses to the final question suggested positivity toward the simulation tool, indicating its potential benefit in terms of providing a range of realistic learning experiences. The responses do suggest that some features of the simulator need to be explained further in the system documentation. The final 3 comments emphasized that the simulator was easy to use and was perceived as a useful addition to the class.

**Table 4:** Poststudy survey of impressions of the hearing screening simulator (n=17).

			Frequency of being mentioned, n (%)
“**What aspects of the simulation did you like?”**			
	Provides hands-on experience, helped me become more familiar with an audiometer, and increased my confidence	5 (29)	
	Ease of use	4 (23)	
	Practice with multiple virtual patients	4 (23)	
	Provided useful feedback on the simulation debriefing report	1 (6)	
	Thought it was helpful	1 (6)	
“**What aspects of the simulator did you not like?”**			
	Did not show how to do otoscopic or tympanometry exam	4 (23)	
	Having better instructions would be helpful	4 (23)	
	Nothing, this is a useful simulation	2 (12)	
	It was repetitive	1 (6)	
“**Do you have any recommendations to improve the simulator?”**			
	The simulator did not show how to perform otoscopy and tympanometry. Clearer instructions are needed in general	5 (29)	
	Nothing, no improvement needed	3 (18)	
	Model the technician giving required directions to the subject	1 (6)	
	Have the design of the simulator match that of the audiometer	1 (6)	
“**Would you like to share anything else?”**			
	A great tool to add to any audiology class	1 (6)	
	Really liked that sample participants showed inconsistencies in hearing thresholds. This realistic touch prepares one to be not thrown off when performing a live, in-person screening	1 (6)	
	The simulation was easy to use	1 (6)	
	The virtual simulation was very helpful	1 (6)	
	I loved the activity!	1 (6)	

### Instructor Evaluation of Student Hearing Screening Assessment Technique

Certified instructors supervised the assessment, observing and grading the students’ performance and technique. The summary data from the instructor’s assessment is given in Table 5. Assessment data for two students in the control group were not available which reduced the size of the control group to 14. Data for one student in the test group was dropped because of incomplete data resulting in a group size of 16. All students in both the control and test groups performed the 3 screenings satisfactorily (ie, otoscopy, tympanometry, and hearing screening). Some students, primarily in the control group, did not follow the prescribed protocol exactly, as reflected in the technique scores shown in Table 5. Students performed similarly across test and control groups, except in the case of providing instructions and screening according to protocol.

**Table 5:** Certified technicians’ assessment of study participants’ techniques in performing otoscopic, tympanometric, and audiometric testing on live subjects using commercial audiometer equipment.

			Control group (n=14), mean (SD)		Test group (n=16), mean (SD)
Otoscope			4.71 (0.47)		4.50 (0.52)
Tympanometer			4.64 (0.74)		4.38 (0.62)
**Audiometer**					
	Provide instructions	3.64 (1.39)		4.63 (0.81)	
	Test according to protocol	4.64 (0.50)		4.81 (0.40)	

## Discussion

### Principal Findings

The HSS is designed as a training tool for speech-language pathologists, nurses, and other professionals to learn the skills needed to perform hearing screening. It can provide remote training when it is not practical to use a traditional practicum setting with commercial audiology equipment, and thus represents an improvement on the state of the art [26]. The design science framework described in this study could be used to replicate and extend the existing simulator and develop other simulation training tools; for example, this implementation is modeled on a specific commercial audiometer. It would be useful to expand this to an array of available audiometers and allow students to receive a flexible range of training on instruments of their choosing. This would be similar to how Microsoft Flight Simulator [29] allows users to choose from 30 different cockpits. During our testing, a respondent commented that the simulation interface did not represent the instrument they were using, highlighting the need to expand the tool to model other instruments. The user training interaction model created for this study incorporated artificial patients in a software simulation within a theoretical framework, operating in real-time and real-world environments. The tool uses external configuration files that enable the addition of new simulated subjects. Each simulated subject has a corresponding biographical sketch, auditory and tympanometric profiles, and otoscopic images. This design feature allows the addition of new simulated patients, covering a range of possible real patient conditions. The software simulation tool provides a design artifact that was tested and evaluated across 33 participants and thus provides a complete design science research cycle, inclusive of artifact evaluation.

This study focused on the user experience that influenced the design parameters of the HSS. In the evaluation, we surveyed users to assess usability, as well as efficacy as a teaching tool. While the mean scores of the test group were largely higher than those of the control group and demonstrated a larger percentage change from before to after the intervention, there were few statistically significant differences between the groups. This was not surprising, considering the very early phase of research in this pilot program and the known difficulties of measuring educational interventions, especially with small sample sizes. Furthermore, the percentage changes from before to after the intervention were mixed for those participants who had previously conducted a screening examination on a real person before participating in this training exercise and study. While the small sample size of this subgroup makes it difficult to draw firm conclusions in this regard, it is important to note that prior screening experience may impact hearing screening learning outcomes and the effect of the simulator on learning. Taken together, the quantitative and qualitative results from this endeavor show significant promise for audiology simulator tools in enhancing the education of speech-language pathology students. The results indicate that the working prototype of the simulator may provide a strong supplement to in-person training using commercially available equipment. Participants noted that the simulator was intuitive and useful for learning the skills needed to perform hearing screenings, while also indicating areas for improvement.

### Limitations

The number of participants in this study was low (n=33), yet appropriate for an initial pilot test. Future studies should include a more diverse set of participants, possibly from multiple geographic locations. This represents the first test of the HSS simulation software. Future iterations of the software may demonstrate different results. A common issue in the control group was a failure of student evaluators to communicate instructions to the subjects as prescribed in the protocol. This issue was not as prevalent in the test group, which may indicate that the added simulator practice impacted the students’ performance in following the procedure, although the simulator did not explicitly indicate or model this behavior. The observed issues were not modeled in the simulation and thus represent potential areas for future improvement.

The study design could be improved. In this iteration, the students in the test group received traditional lectures on the material and then completed the simulation before their performance and technique were evaluated. The control group received only the traditional lectures before evaluation. The difference in the observed results could therefore be due to the test group’s greater exposure to the relevant content. In future testing, the control group could be given an alternative, related activity with a similar duration as the simulation. This could include additional reading or researching audiometer test equipment on the internet.

### Future Directions

Our testing of the HSS found that it has the potential to be an effective audiometry teaching tool, but some areas for improvement and future research directions were identified to validate these findings. The most significant improvement would be to convert it to a web-based platform for broader availability and accessibility. While remote students can currently use the simulator, it requires them to download and install the tool locally. A web-based option would make using the simulator easier, with the only requirement being a web browser.

The functionality of the simulator could be extended by allowing the configuration to support customizable pass and refer cutoffs to address different environments or countries. Similarly, allowing the configuration to support multiple languages could be valuable. This could be implemented by modifying and displaying text in different user-specified languages or incorporating “tool tips” to offer second-language support in pop-up windows.

Another important extension would be the inclusion of different models of commercial audiometer test equipment. A key benefit of the tool is that it provides training using a digital twin, providing the learning experience with a test unit user interface. The value of this feature diminishes if the technician is not using the same equipment modeled in the tool. This issue was mentioned by a participant in the exit survey. Although the tool is designed for hearing screening test training, it could be extended to handle diagnostic hearing test training as well. While the physical equipment remains the same, the testing protocols differ and would have to be modified.

Recommendations from students using the system included that a video tutorial should be created to either replace or supplement the current text-based instructions. The students also suggested adding a learning mode, where context-sensitive feedback could be displayed on command during the training phase but hidden during the evaluation phase. As a result of the testing, it was determined that the simulator did not verify the students’ compliance with the required auditory screening protocols. While the system counts the number of frequency screens completed, it does not track the testing of specified frequencies (1000, 2000, and 4000 Hz tones), repeated tests, the number of positive responses, or the sound levels used. Therefore, while the simulator did provide an assessment of the students’ ability to determine the correct result, it did not fully capture the performance as specified in the audiology screening guidelines [4]. These factors were considered by the evaluator when grading the students during the evaluation phase of the exercise. These features are planned for future versions of the simulator. To further access the effectiveness of the simulator tool, a series of studies would need to be conducted with a broader set of participants across a broader set of training programs (eg, universities) dispersed geographically. These studies would need to assess the educational outcomes associated with the use of the tool.

### Conclusions

This work’s contributions include a design framework for developing a tool that simulates an existing process, the description of a unique artifact that supports an individualized and self-paced learning environment incorporating context-sensitive feedback and performance assessment, and an extensible approach to supporting simulated subjects in clinical training testing artificial patients. The software development process, developed artifact, and user evaluation together provide a comprehensive, descriptive framework for designing audiology simulation training tools as well as validation that such tools may provide a cost-effective mechanism for training a wide range of providers for audiology screening.

## Abbreviations

HSS: hearing screening simulator

## Disclaimer

An earlier version of this paper was presented at the 55th Hawaii International Conference on System Sciences [28]. This paper differs from the earlier conference paper. The discussion of the iterative design of the simulator tool was removed, with interested readers directed to the conference paper. This study changed statistical methodology from the Kruskal-Wallis test to the Mann-Whitney U test to more appropriately address the study’s pretest-posttest design with ordinal data. Statistical reporting was expanded to include *P*<.05 and *P*<.10, both of which are often used for reporting in pilot studies. Additional mean change and percentage change analyses were conducted and reported on regarding the prior audiology testing experience of participants. A new table was added to present the additional analysis. Corrections were made throughout the paper, with significant reduction and rewriting of the introduction and literature review. The discussion section was expanded based on the expanded statistical analysis.

## Data Availability

The datasets generated and analyzed during this study are available in the OSF repository “Hearing Screening Simulator Data” [30] under the Creative Commons Attribution 4.0 International Public License.
